# Evaluating the Reliability of a Shape Capturing Process for Transradial Residual Limb Using a Non-Contact Scanner

**DOI:** 10.3390/s22186863

**Published:** 2022-09-10

**Authors:** Calvin C. Ngan, Harry Sivasambu, Sandra Ramdial, Jan Andrysek

**Affiliations:** 1 Institute of Biomedical Engineering, University of Toronto, Toronto, ON M5S 3G9, Canada; 2 Bloorview Research Institute, Holland Bloorview Kids Rehabilitation Hospital, Toronto, ON M4G 1R8, Canada

**Keywords:** digital technology, prosthetics, orthotics, 3D scanning, transradial, amputees, residual limb

## Abstract

Advancements in digital imaging technologies hold the potential to transform prosthetic and orthotic practices. Non-contact optical scanners can capture the shape of the residual limb quickly, accurately, and reliably. However, their suitability in clinical practice, particularly for the transradial (below-elbow) residual limb, is unknown. This project aimed to evaluate the reliability of an optical scanner-based shape capture process for transradial residual limbs related to volumetric measurements and shape assessment in a clinical setting. A dedicated setup for digitally shape capturing transradial residual limbs was developed, addressing challenges with scanning of small residual limb size and aspects such as positioning and patient movement. Two observers performed three measurements each on 15 participants with transradial-level limb absence. Overall, the developed shape capture process was found to be highly repeatable, with excellent intra- and inter-rater reliability that was comparable to the scanning of residual limb cast models. Future work in this area should compare the differences between residual limb shapes captured through digital and manual methods.

## 1. Introduction

Digital technology, such as computer-aided design/computer-aided manufacturing (CAD/CAM) systems, was introduced to the field of prosthetics more than three decades ago. The applications of CAD come in various forms, including digital shape capture systems, digital rectification software, computer-guided carving machines, and additive manufacturing. In recent years, various optical non-contact scanning systems have been developed [1,2,3,4]. In particular, structured light scanners are one of the most commonly used systems. It acquires the surface geometry of the object of interest by illuminating a sequence of visible light patterns and using a camera to measure the distortions in the light pattern over the object. The degree of distortion at each point corresponds to the distance between the object and the camera. Ultimately, a collection of coordinates (x, y, z) in space, named point cloud, is generated and reconstructed as a 3D model. In the context of prosthetics, the 3D residual limb model is useful for clinical assessment as it provides quantitative information about the shape of the residual limb. This allows clinicians to closely monitor the changes in residual limb shape and volume to maintain an accurate and comfortable fit of the prosthetic socket, especially in the immediate postoperative period [5,6].

Studies have evaluated the reliability and validity of capturing transtibial and transfemoral residual limb cast (static) models with structured light scanners. They found that structured light scanners demonstrated a high level of accuracy and reproducibility, signifying their potential to be applied in clinical practice to collect quantitative records, either for comparative study or data storage [2,3,4,7]. Recent studies have also investigated the use of non-contact scanners directly on the lower limb residuum and compared it to traditional clinical practices, such as circumferential measurements and casting [8,9]. Greater reliability was observed for the use of non-contact scanners in both studies.

However, previous studies have primarily assessed scanner performance on lower limbs and not upper limbs [2,3,4,7,8,9,10]. The inherent size and volume differences between upper and lower residual limbs could impact the performance of the scanners. In addition, unlike residual lower limbs and traditional casting methods [8,11], to the best of the authors’ knowledge, there are no guidelines for digitally capturing the shape of residual upper limbs using a non-contact optical scanner, particularly for the transradial population. Yet, it is known that a standardized positioning method for the residual limb is critical in ensuring that the data points defining the shape are recorded in a geometrically regular manner [12,13]. Therefore, the objective of this study was to develop an optical scanner-based shape capture process for direct scanning of transradial residual limbs and assess the reliability of its volumetric and shape measurements.

## 2. Materials and Methods

### 2.1. Shape Capturing Process

#### 2.1.1. Setup

**Figure 1:**
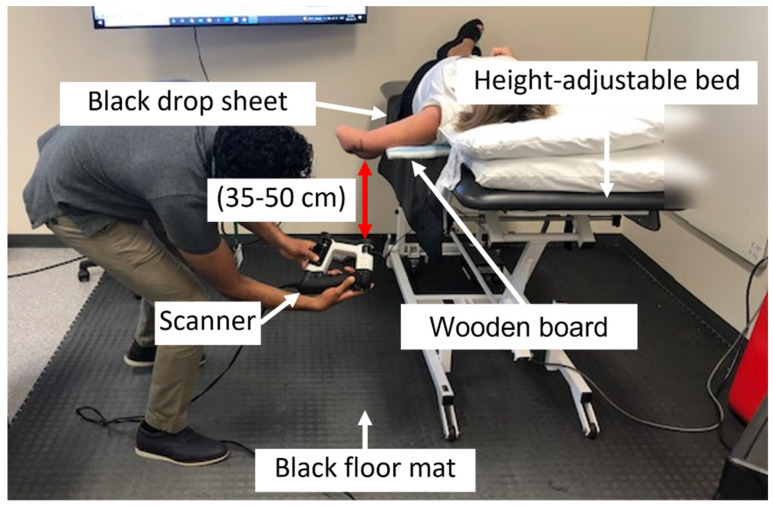
A description of the main aspects of the shape capture setup showing the client (on the height-adjustable bed) and the individual performing the scanning. Noteworthy are the features (sheet and mat) to improve scanner performance, as well as the positioning of the client’s arm using a thin wooden board.

The setup of the designated scanning area is shown in Figure 1. Fluorescence lights are the source of ambient lighting. Sunlight from windows is blocked. The use of a height-adjustable bed allows for sufficient clearance to maintain a 35–50 cm distance between scanner and posterior aspect of the limb. Black floormats and drop sheets are used to improve contrast between the residual limb and the background, resulting in reduced noise and artifacts in the scan data. A thin wooden board (200 mm × 760 mm × 12 mm) is used to support client’s shoulder and upper arm to stabilize their residual limb and minimize movement and fatigue during shape capture.

#### 2.1.2. Instrument

The study used the Spectra scanner developed by Vorum (Vancouver, BC, Canada) [14]. It is a handheld 3D structured light scanner with a resolution of 0.1mm. During operation, the Spectra scanner captures 15 photographs per second. All captured images are imported into the Spectra software (Vorum, Vancouver, BC, Canada) in real time to create a 3D view of the model. The Spectra scanner was selected due to its availability at where this study was conducted (Holland Bloorview Kids Rehabilitation Hospital, Toronto, ON, Canada). Other commercially available structured light scanners in the field of prosthetics, including the Omega (structured light) scanner (Ohio Willow Wood, Mt. Sterling, OH, USA) and Artec Eva (Artec Group, Luxembourg) would also be ideal alternatives as they are considered as precise, accurate, and suitable for clinical use [2,4,7].

#### 2.1.3. Shape Capture Procedure

**Figure 2:**
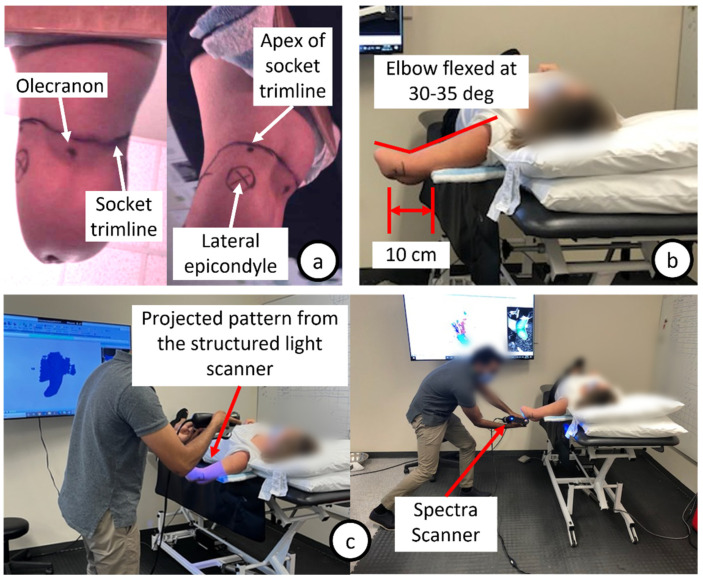
(**a**) Key anatomical landmarks identified and marked by the treating prosthetist; (**b**) The limb absentee is instructed to lay supine on a height-adjustable bed with their residual limb shoulder placed onto a thin wooden board; (**c**) The scanner is aimed at the residual limb directly above its surface., then moved in a steady and continuous manner either clockwise or counterclockwise around the residual limb.

Prior to shape capture, the prosthetist performed a physical examination of the residual limb to assess strength, range of motion, and skin condition. Using an indelible pencil, the prosthetist marked key anatomical landmarks and other areas such as bony prominences, sensitive regions, and myoelectric sites depending on their particular practice, as well as the trimline of the socket (Figure 2a). Traditionally, these areas of interest on the upper limb are marked to guide prosthetists during traditional rectification to aid the design process and improve likelihood of an appropriate socket fit and pressure distribution [15].

Subsequently, with the residual limb arm exposed up to the shoulder (i.e., clothing removed), the limb absentee was instructed to lay supine on a height-adjustable bed with their residual limb shoulder placed onto a wooden board. The elbow should be at least 10 cm away from the edge of the wooden board to allow for full capture of the residuum. Then the limb absentee was instructed to flex their elbow to approximately 30–35 degrees with the aid of a goniometer, as it is a standard practice during traditional shape capture [16], and remained as still as possible for the duration of each scan (Figure 2b). A stockinette can be donned to cover the arm and aid with the consistency of the skin tone and skin condition of the client during scanning. To start scanning, the scanner was aimed at the anterior surface of the residual limb. Then the scanner was moved in a steady and continuous manner either clockwise or counterclockwise around the residual limb. The key is to keep the scanner perpendicular to the surface of the residual limb whilst maximizing the amount of light projected onto the limb (Figure 2c). Each scan was 30 to 60 s in duration.

### 2.2. Evaluation of Reliability

#### 2.2.1. Participants

In this study, participants with an upper limb absence at the transradial level (below elbow) were recruited from a list of clients identified by prosthetists from the Holland Bloorview Kids Rehabilitation Hospital (Toronto, ON, Canada). Clients were included onto the list by prosthetists if they satisfied the following inclusion criteria: (1) has had a transradial limb absence for at least 2 years and (2) can hold their residual limb in the same position for at least one minute.

#### 2.2.2. Data Collection

Participants attended a single digital shape capture session. The digital shape capturing procedures were performed by two independent observers in randomized order. The observers were researchers with about 5 h of formal training (provide by Vorum) and familiarization with the scanner and scanning protocols (prior to data collection).

To evaluate the reliability of the digital shape capturing process, each observer repeated the digital shape capture process three times. This resulted in a set of six scanned residuum models (two observers × three repetitions) per participant. Relevant health information, such as sex, age, and cause of limb absence were also collected. The study was approved by the Research Ethics Board at Holland Bloorview Kids Rehabilitation Hospital (Toronto, ON, Canada).

#### 2.2.3. Data Processing

The reliability of the digital scanning process was evaluated in terms of the total volume of each residual limb model, as well as its geometric profiles such as the overall Anterior-Posterior (A-P) and Medial-Lateral (M-L) measurement.

**Figure 3:**
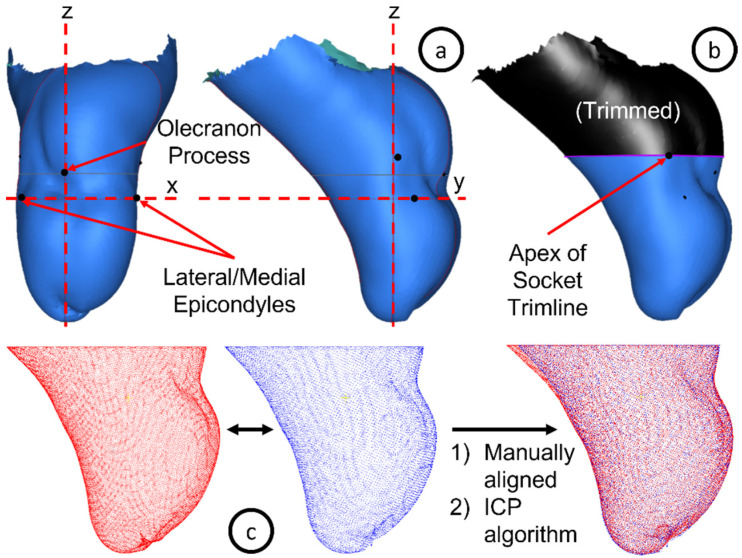
(**a**) Key anatomical landmarks were used to define the XYZ coordinate system, where the X-axis is defined by the medial and lateral epicondyles, the Z-axis would pass through the distal end of the model, and the Y-Z plane is aligned with the olecranon process; (**b**) Volume of interest is defined and extracted; (**c**) Models are overlayed and manually aligned using the graphical user interface in the Spectra Software, then their alignment is refined with an iterative closest point (ICP) algorithm.

All models were post-processed using the Spectra and Canfit O&P CAD software (Vorum, Vancouver, BC, Canada) and saved as an “STL” file. Each set of six residual limb models collected from each participant was processed independently. Anatomical landmarks and features were labelled on each set of residuum models based on colour information overlayed on the scanned image. In order to align and compare the residual limb models, a coordinate convention was developed based on the key anatomical landmarks and features, including the olecranon process, and lateral and medial epicondyles (Figure 3). The boundary of the volume of interest was defined by a cutting plane that was parallel to the X-Y plane and through the marking on the apex of the socket trimline. The selected volume of interest resembled the portion of residual limb that is typically casted during traditional shape capture for a transradial socket [16]. An iterative closest point algorithm was then used to refine the alignment of these models.

**Figure 4:**
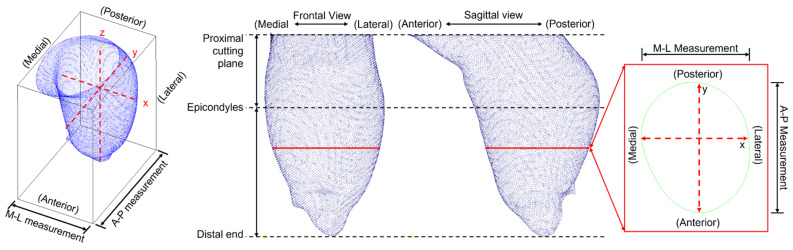
Each residual limb model is confined in a bounding box in the software. Then each model was sliced in the X-Y plane along the Z-axis. A slice is as shown in the red box. The cross-section area (CSA), Medial-Lateral (M-L) and Anterior-Posterior (A-P) measurement of each slice were also computed.

The volume of each model from the distal end to the proximal cutting plane was calculated using Meshmixer 3.5 (Autodesk, Inc., San Rafael, CA, USA). With regard to the geometric profiles of the residuum model, each set of models was first imported to CloudCompare 2.11, an open-source 3D point-cloud processing software. The overall residual limb sizes along the X-axis (i.e., overall M-L measurement) and Y-axis (i.e., overall A-P measurement) were computed using the bounding box encompassing the entire model [4]. In addition, each model was separated into two regions, below-the-elbow (distal end to epicondyles) and above-the-elbow (epicondyles to proximal cutting plane). Each region was then sliced in the X-Y plane along the Z-axis from the at intervals of 1% (Figure 4). CSA, M-L and A-P measurement of each slice were then computed using Matlab (The MathWorks, Inc., Natick, MA, USA) [4,7].

#### 2.2.4. Statistical Analysis

Intra- and inter-rater reliability of the digital scanning process in terms of volumetric measurements, overall M-L and A-P measurements were assessed. Descriptive statistics including the mean difference and standard deviation values were calculated. For intra- and inter-rater reliability, interclass correlation coefficients (ICC) were calculated using the ICC (2,1) equations with a 95% confidence interval (CI). A threshold of ICC > 0.90 was selected for the level of reliability [3,4]. In addition, Bland–Altman plots were used to assess the agreement between observers on volumetric measurement [3,4,7,17]. In order to detect an ICC of 0.9, power of 80% and alpha of 0.05, a sample size of 15 participants was used [18]. In order to further examine the differences in shape measurement, the mean differences within and between observers in CSA, A-P and M-L measurements of each slice along the length of the residuum model were also computed [4,19].

## 3. Results

**Figure 5:**
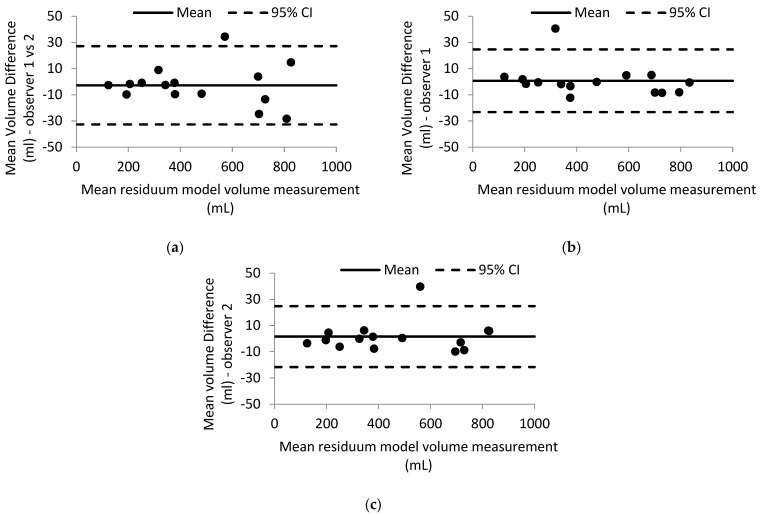
(**a**) Bland-Altman plot of mean volume difference of residuum models (**a**) between observers 1 and 2, (**b**) between repeated measurements by observer 1; and (**c**) between repeated measurements by observer 2.

In total, 15 participants were recruited (Table 1) between September 2020 to December 2021. The assessment of reliability showed high levels of intra- and inter-rater reliability on volume measurements, with all ICCs ranging from 0.998 to 0.999. In all, 95% CIs ranged between 0.995 and 1 (Table 2). Bland–Altman plots of the reliability analysis demonstrated good agreement within and between observers (Figure 5), with only one out of the 15 repeated mean differences being outside of the standard deviation limits.

Regarding the shape measurement, there were high levels of intra- and inter-rater reliability on the overall M-L measurement of the residuum model, with all ICCs ranging from 0.991 to 0.996 and 95% CIs ranged between 0.981 and 0.998 (Table 3). ICCs for the overall A-P measurement of the model ranged from 0.918 to 0.946, but their 95% CIs ranged between 0.832 and 0.980, that is, the lower bound of the CIs was lower than the selected threshold of 0.90.

**Figure 6:**
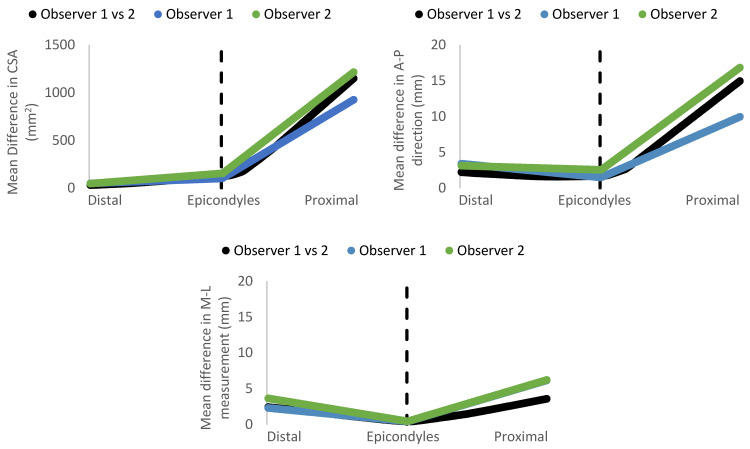
Mean differences in CSA, A-P and M-L measurements within and between observers along the residuum model across 15 participants. The residuum lengths were normalized for each participant to match up the distal and proximal ends, as well as the epicondyles. The dash line indicates the location of the epicondyles.

To further examine the differences in shape measurement within and between observers, Figure 6 revealed that the mean differences in CSAs and A-P measurements along the residuum model shared a similar trend, where the discrepancies were always higher in the region between the epicondyles to the proximal portion compared to the region between distal end to epicondyles, both within and between observers. In contrast, the mean measurement differences in the M-L direction between the two regions were similar.

## 4. Discussion

Past research related to the digital capture of residual limb shapes has been limited to static models and mostly focused on the lower limbs. This paper uniquely evaluated the reliability of a digital shape capture process for transradial residual limbs based on direct scans of the limb. The main findings indicate that transradial limb scans can be acquired reliably, and on par with static models. As discussed in detail below, the main source of variability in residual limb scans stems from the positioning and alignment of the limb during scanning; hence underscoring the importance of establishing clinically viable scanning procedures. In this regard, this paper describes a scanning process and setup that was developed as part of this project. Given the paucity of literature and clinical best practices, the process (described in Section 2.1) may provide an important foundation for the clinical utilization of digital workflows for upper limb prosthetic applications.

Several studies examined the reliability of using 3D scanners to capture the shape and volume of transtibial and transfemoral cast models (static objects) as well as directly on lower limb residuum. Dickinson et al. [7] evaluated the Go!SCAN 3D scanner (Creaform Inc., Levis, QC, Canada) with 20 transtibial limb cast models and reported excellent intra- and interrater reliability, both ICCs exceeding 0.996 (95% CIs: 0.990–0.999). A follow-up study with 11 participants with transtibial amputation yielded similar results [9]. Seminati et al. [4] assessed the reliability of the Artec Eva Scanner (Artec Group, Luxembourg, Luxembourg) on five (*n* = 5) transfemoral and five (*n* = 5) transtibial cast models with ICCs of 0.99 for both intra- and interrater reliability. The same research team has conducted a similar study recently, in which five (*n* = 5) transtibial and five (*n* = 5) transfemoral residual limbs were scanned directly, and the reported ICCs were between 0.995–0.998 (95% CIs: 0.990–0.999) [8].

Scanning directly the person’s limb and eliminating the need for physical casting, is a more efficient process that enables a fully digital clinical workflow. There are two commonly listed concerns with direct limb scans. Firstly, patient movement and shifting of the limb during scanning may lead to inadvertent muscle contractions and joint and tissue movement that could result in distortion or failure of the scan [10]. Secondly, design and performance limitations of existing scanners makes it challenging to capture smaller residual limbs including transradial residuum [20]. To a degree, both concerns have been targeted through the scanning protocol detailed in Section 2.1, developed and optimized by our team. Overall, high levels of intra- and inter-rater reliability in total residual volume measurements were reported in this study, with all ICCs comparable to previous studies with cast models and exceeding 0.998 and 95% CIs ranged between 0.995 and 1. The high intra- and inter-rater reliability suggests the scanning process has excellent reliability in volumetric measurement and that inadvertent movement of the limb can be controlled through proper positioning and supporting of the patient. In addition, previous reliability studies measured transtibial and transfemoral cast models ranging in volume between 918 and 2497 mL [2,4,21]. In comparison, the mean volume of the residuum models in this study were considerably smaller (467 ± 238 mL). This suggests that the developed scanning protocol can be used to capture small residual limbs without compromising the reliability of the volumetric measurements. Lastly, the overall mean volume difference between observers measured across all scans was −2.75 ± 15.27 mL, which equates to 2.68–3.86% of the mean residual model volume. According to works on lower-limb amputation from Sanders et al. [22], a 6% change in residual limb volume may cause clinically significant changes to quality of fit, comfort, and device satisfaction. Although similar work has not been recreated for upper-limb amputation, the sub-4% values found in our study provided reasonable confidence about the adequacy of the scanning protocol, and ultimately the ability to design well-fitting sockets.

In terms of shape measurement, excellent levels of intra- and inter-rater reliability in overall M-L measurements were reported in this study, with all ICCs ranged between 0.991 and 0.996 with 95% CIs ranged from 0.981 to 0.998. These results were similar to reported ICCs of 0.99 [4] and 0.99 (95% CIs: 0.99–1.00) [7] in previous studies with cast models. In comparison, the reported ICCs for the intra- and inter-rater reliability in overall A-P measurements ranged from 0.918 to 0.946, but their 95% CIs were ranged between 0.832 and 0.980. Thus the range of 95% CIs were too wide to conclude that the overall A-P measurement was reliable as a minimum level of 0.90 of ICC was needed clinically [3,4]. Though this discrepancy could be explained when examining the CSA analysis (Figure 6). CSA analysis was performed using axial profiles along the length of the model. These results showed a sharp increase in mean difference towards the most proximal end of the models (i.e., above the epicondyles). Upon further analysis, A-P measurement errors disproportionately contributed to the error compared to M-L measurements. The authors speculated that this error originated from slight differences in elbow flexion between scans, and thus would not affect the clinically relevant portion of the residual limb (below the epicondyles). In addition, this error would not impact the prosthetist’s ability to use digital scans to design transradial sockets since transradial sockets are designed to maximize the range of motion of the elbow [23,24]. This is achieved by contouring the anterior part of the socket below the cubital fossa. Furthermore, transradial sockets are securely attached and suspended to the residual limb by constricting the socket over the medial and lateral epicondyles [25,26], thus minimizing the importance of the A-P measurement at and proximal to the elbow.

The main limitation of the study was that only one scanner, and two observers were used to collect data. Future studies may consider repeating the protocol with different non-contact optical scanners. In addition, to compare the residuum models, they had to be manually aligned in the post-processing phase. Although an iterative closest point (ICP) algorithm was used to refine the alignment, some measurement errors could potentially be introduced due to model misalignment. Lastly, unlike the traditional casting process where the clinician can use plaster bandages to adjust the shape and apply pressure where needed, the scanned limb is uncompressed and therefore the scanned model may be a more difficult starting point to design the socket [9,20]. A direct comparison of limb shapes acquired using casting versus scanning remains an investigation for future work.

## 5. Conclusions

This study developed and tested a protocol to digitally capture the shape of transradial amputee residual limbs directly using a non-contact optical scanner. These findings show the feasibility of using non-contact scanners for the quantification of the shape and volume of transradial residual limbs, and possibly for the design and fabrication of prosthetic sockets and devices. Future work in this area should compare the differences between residual limb shapes captured through digital and manual methods.

## Figures and Tables

**Table 1:** Participant Characteristics. Participants were listed chronologically based on the date of shape capture appointment.

Participant	Sex	Cause of Limb Absence	Side	Age (Years)	Residuum Model Volume (mL)	Residuum Model Length (mm)	Overall A-P Measurement (mm)	Overall M-L Measurement (mm)
1	M	Congenital	L	9	571	141	140	84
2	M	Congenital	L	32	703	235	126	81
3	M	Trauma	R	38	809	280	124	78
4	F	Congenital	R	9	123	59	65	61
5	F	Congenital	L	59	482	118	114	90
6	F	Congenital	R	62	380	94	117	88
7	M	Congenital	L	40	378	94	92	78
8	M	Congenital	L	5	193	100	92	59
9	F	Congenital	L	11	252	89	111	69
10	F	Congenital	L	22	206	89	95	63
11	F	Congenital	L	14	726	123	129	96
12	M	Congenital	L	17	343	94	82	79
13	M	Trauma	R	13	699	127	130	105
14	M	Congenital	L	43	825	252	123	86
15	M	Congenital	L	14	316	83	140	79
Mean (1 SD)				24.6 (18.7)	467 (238)	132 (68)	112 (22)	80 (13)

**Table 2:** Intra-rater and inter-rater reliability of volume measurements.

	Observer	MD (SD)	ICC	95% CI
Intra-rater	1	0.73 (12.21)	0.999	0.998–1.000
	2	1.60 (11.86)	0.998	0.996–0.999
Inter-rater	1 vs. 2	−2.75 (15.27)	0.998	0.995–0.999

MD: mean difference between volume calculations (mL); SD: one standard deviation (mL); ICC: intraclass correlation coefficient; 95% CI: 95% confidence interval for ICC.

**Table 3:** Intra-rater and inter-rater reliability of shape measurements.

	Observer	MD (SD)	ICC	95% CI
Overall A-P measurement				
Intra-rater	1	0.49 (4.16)	0.946	0.880–0.980
	2	0.88 (4.35)	0.926	0.832–0.973
Inter-rater	1 vs. 2	−0.05 (7.71)	0.918	0.841–0.969
Overall M-L measurement				
Intra-rater	1	−0.34 (0.99)	0.996	0.990–0.998
	2	0.09 (1.38)	0.992	0.981–0.997
Inter-rater	1 vs. 2	−0.80 (1.32)	0.991	0.981–0.997

MD: mean difference between profile measurements (mm); SD: one standard deviation (mm); ICC: intraclass correlation coefficient; 95% CI: 95% confidence interview for ICC.

## Data Availability

Not applicable.
